# Reduced Efficacy of Praziquantel Against *Schistosoma mansoni* Is Associated With Multiple Rounds of Mass Drug Administration

**DOI:** 10.1093/cid/ciw506

**Published:** 2016-07-28

**Authors:** Thomas Crellen, Martin Walker, Poppy H. L. Lamberton, Narcis B. Kabatereine, Edridah M. Tukahebwa, James A. Cotton, Joanne P. Webster

**Affiliations:** 1 Department of Infectious Disease Epidemiology and the London Centre for Neglected Tropical Disease Research, Imperial College London, St Mary's Campus; 2 Wellcome Trust Sanger Institute, Hinxton; 3 Department of Pathology and Pathogen Biology, Royal Veterinary College, University of London, Hertfordshire; 4 Institute of Biodiversity, Animal Health & Comparative Medicine and Wellcome Trust Centre for Molecular Parasitology, University of Glasgow, United Kingdom; 5 Division of Vector Bourne Diseases, Ministry of Health, Kampala, Uganda

**Keywords:** parasite, schistosomiasis, praziquantel, generalized linear mixed model, anthelmintic efficacy

## Abstract

***Background.*** Mass drug administration (MDA) with praziquantel is the cornerstone of schistosomiasis control in sub-Saharan Africa. The effectiveness of this strategy is dependent on the continued high efficacy of praziquantel; however, drug efficacy is rarely monitored using appropriate statistical approaches that can detect early signs of wane.

***Methods.*** We conducted a repeated cross-sectional study, examining children infected with *Schistosoma mansoni* from 6 schools in Uganda that had previously received between 1 and 9 rounds of MDA with praziquantel. We collected up to 12 *S. mansoni* egg counts from 414 children aged 6–12 years before and 25–27 days after treatment with praziquantel. We estimated individual patient egg reduction rates (ERRs) using a statistical model to explore the influence of covariates, including the number of prior MDA rounds.

***Results.*** The average ERR among children within schools that had received 8 or 9 previous rounds of MDA (95% Bayesian credible interval [BCI], 88.23%–93.64%) was statistically significantly lower than the average in schools that had received 5 rounds (95% BCI, 96.13%–99.08%) or 1 round (95% BCI, 95.51%–98.96%) of MDA. We estimate that 5.11%, 4.55%, and 16.42% of children from schools that had received 1, 5, and 8–9 rounds of MDA, respectively, had ERRs below the 90% threshold of optimal praziquantel efficacy set by the World Health Organization.

***Conclusions.*** The reduced efficacy of praziquantel in schools with a higher exposure to MDA may pose a threat to the effectiveness of schistosomiasis control programs. We call for the efficacy of anthelmintic drugs used in MDA to be closely monitored.

## Teaser

The efficacy of praziquantel against *Schistosoma mansoni* was significantly lower in Ugandan schools that had received more prior rounds of mass drug administration, as determined by fitting a statistical model to parasite egg counts before and after treatment.

*Schistosoma mansoni* is a parasitic trematode and an etiological agent of the neglected tropical disease schistosomiasis, which is estimated to infect >230 million people worldwide [1]. The parasite has a complex life cycle involving a snail intermediate host, with humans becoming infected through skin contact with infectious water sources. Chronic morbidity of intestinal schistosomiasis is caused by eggs from adult *S. mansoni* that become trapped in tissues. This leads to granuloma formation that can cause fibrosis and portal hypertension [2]. Conservative estimates of morbidity indicate that *S. mansoni* infection accounts for 4.4 million cases of blood in stool and 8.5 million cases of hepatomegaly in sub-Saharan Africa [3].

Interventions to control schistosomiasis have varied over time. Early national control programs, such as the Sudanese Blue Nile Health Project (started in 1979) and the Egyptian national program (started in 1988), used a combination of chemotherapy, snail control, provision of clean water and sanitation, and infrastructural development [4, 5]. Such multiapproach control programs are now the exception rather than the rule [6]. Due to the low cost, safety, and high efficacy of praziquantel, mass drug administration (MDA) has become the mainstay of national control programs.

MDA began in Uganda in 2003 and 400 000 treatments of praziquantel were distributed that year, with the support of the Schistosomiasis Control Initiative [7–9]. This has since expanded to the treatment of approximately 1.5 million children annually [10], resulting in significant reductions in *S. mansoni* prevalence, intensity, and associated morbidity [11, 12]. Similar successes have been reported in other countries implementing MDA [13]. Spurred by these results, the World Health Organization (WHO) revised its strategic plan from disease control to the interruption of transmission in certain African countries, including Uganda, by 2025 [14]. With the exception of certain islands implementing snail control [15], this ambitious plan remains solely reliant on MDA with praziquantel. Monitoring the continued efficacy of praziquantel is therefore paramount.

In the field, *S. mansoni* is commonly detected by the presence of eggs in stool, typically using the Kato-Katz thick smear method, which is inexpensive and practical in low-resource settings [16]. Because the test suffers from poor sensitivity, though close to 100% specificity [16], performing multiple readings improves the chances of detecting eggs in the stools of infected patients [17]. Egg counts measured before and after treatment are used to quantify drug efficacy as the egg reduction rate (ERR). The WHO has set a tentative ERR threshold of 90% as an indicator of optimal efficacy [18].

The sample ERR is typically calculated directly from egg count data by applying a simple formula (see equation 1 in “Methods” section). This is a population-level approach that is not readily compatible with individual patient data [19]. Such data are better analyzed using statistical techniques specifically designed for longitudinally repeated measures and that permit estimation of ERRs (and their associated uncertainties) for individual patients [20]. These methods can also be used to estimate distributions of ERRs from individual patients, which are useful for identifying poor responses and gradual shifts in anthelmintic efficacy [19, 20].

Here we assess the effect of previous rounds of MDA on the efficacy of praziquantel against *S. mansoni* infections in primary schoolchildren in eastern Uganda. We explore whether the number of previous rounds of MDA is associated with efficacy of praziquantel, estimated as an ERR. We analyze data from a repeated cross-sectional study of 6 schools that have received varying numbers of MDA rounds over a period of 11 years. We fit a statistical model to data on schistosome egg counts before and 25–27 days after treatment, estimating praziquantel efficacy using Bayesian techniques at the levels of individual patients and schools.

## METHODS

### Study Site

**Figure 1.**
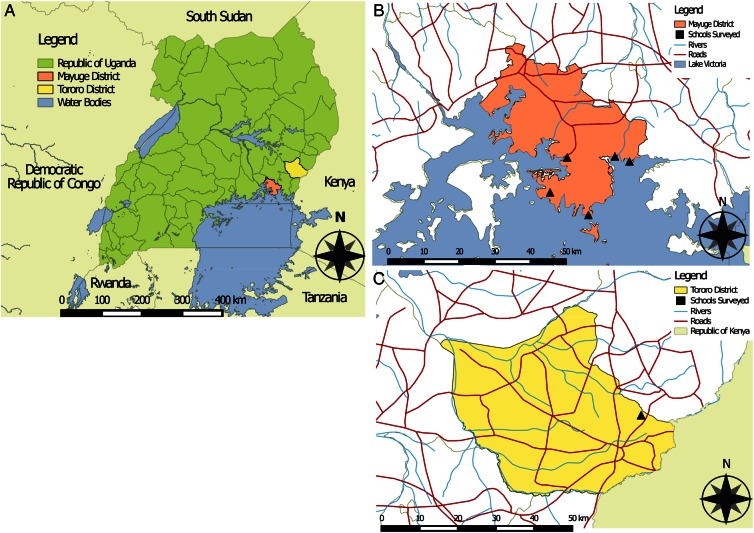
Maps of study sites. *A*, Map of Uganda in an East African context with Mayuge and Tororo districts highlighted. *B*, Location of the schools (triangles) surveyed in Mayuge district. *C*, Location of the school (triangle) surveyed in Tororo district.

Fieldwork was conducted in Mayuge and Tororo districts, Uganda, from May to July 2014 (Figure 1). Six government-run primary schools were included in the study, 5 from Mayuge district and 1 from Tororo (Table 1; see Supplementary Materials for GPS coordinates). None of the schools had piped running water or a pump, and all sanitation facilities were drop pits. Approximately 6 months had elapsed between the previous round of MDA and the present study taking place. The MDA in Tororo in 2013 was the first MDA in this district and achieved only 21% coverage. MDA in Mayuge has been ongoing since 2003, although MDA did not take place in any schools during 2008–2009.

**Table 1.** Sample Estimates of the Prevalence and Intensity of *Schistosoma mansoni* Infection in Children From 6 Primary Schools in Eastern Uganda Before Treatment With Praziquantel

School	District	MDA Exposure Category	Recruited, No.	Tested at Baseline, No.	Positive for *S. mansoni*, No. (%)	Mean EPG (95% CI)
Bwondha	Mayuge	High (9 rounds)	98	96	87 (90.63)	742 (518–973)
Bugoto	Mayuge	High (9 rounds)	183	170	144 (84.71)	436 (339–534)
Musubi	Mayuge	High (8 rounds)	128	127	120 (94.49)	465 (362–600)
Bukoba	Mayuge	Medium (5 rounds)^a^	120	118	53 (44.92)	92 (67–128)
Bukagabo	Mayuge	Medium (5 rounds)^a^	120	118	57 (48.31)	306 (197–442)
Kocoge	Tororo	Low (1 round)	120	120	81 (67.50)	347 (245–456)
Total			769	749	542 (72.36)	382 (338–446)

The number of previous rounds of MDA to which the school has been exposed and corresponding MDA exposure categories are shown. Infection intensity is quantified as the arithmetic mean number of EPG. Confidence intervals are calculated using a block bootstrap approach to account for correlation among egg counts repeatedly measured from the same child.

Abbreviations: CI, confidence interval; EPG, egg per gram of feces; MDA, mass drug administration.

^a^ Estimated from district-level MDA coverage from Ministry of Health.

### Enrollment

**Figure 2.**
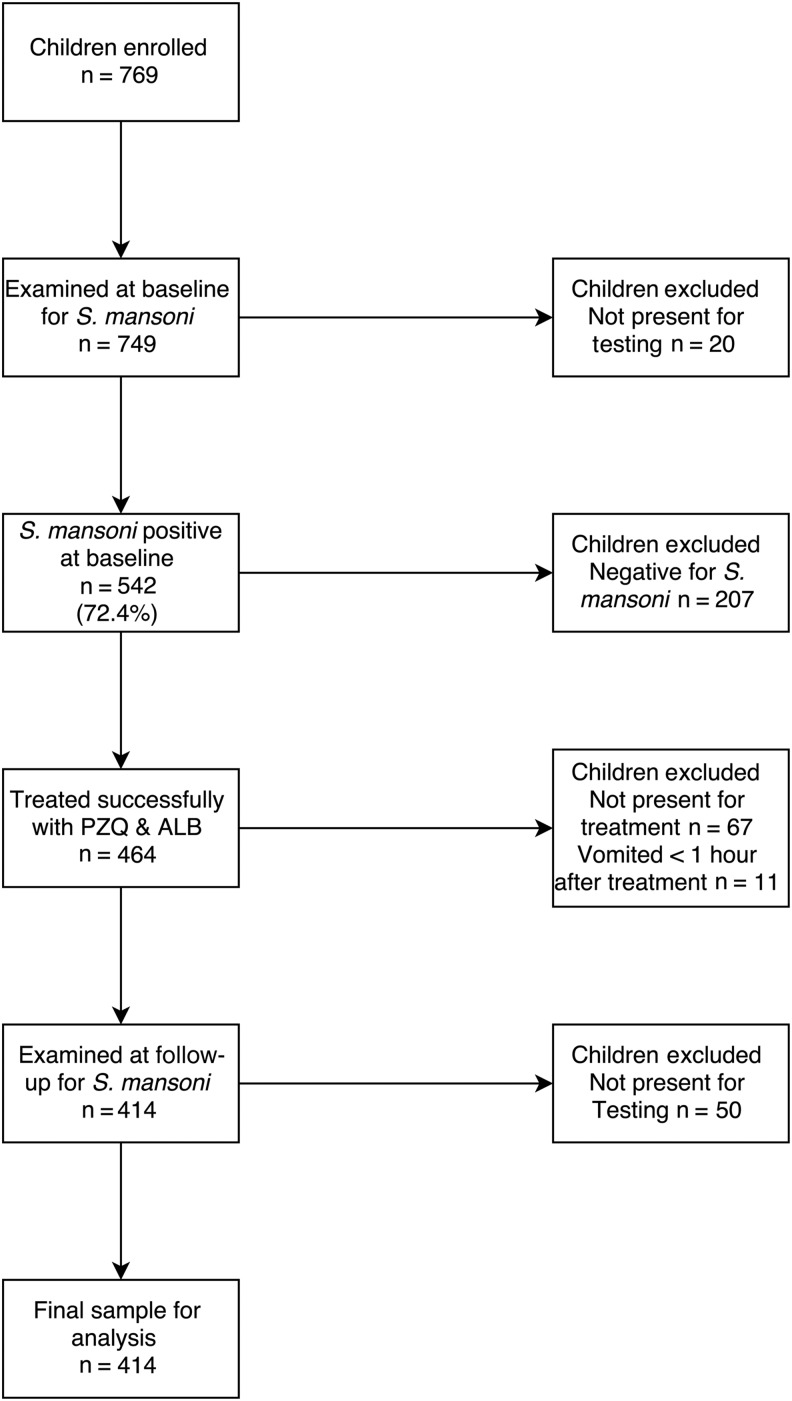
Flow diagram of patient recruitment and exclusion criteria for egg reduction rate analysis after treatment with praziquantel (PZQ) and albendazole (ALB).

At baseline, 769 children were recruited from 6 primary schools. Children were randomly sampled within the age group 6–12 years and by sex. Children enrolled in the study were examined from 1 to 3 days before and 1 to 3 days after treatment with duplicate Kato-Katz slides per stool per day (ie, a maximum of 12 counts per child, up to 6 pretreatment and 6 posttreatment). Treatment with praziquantel and albendazole, for soil-transmitted helminths (STHs), was overseen by a nurse, and children were observed for 1 hour following treatment to check for side effects and to exclude from the efficacy analysis any children who vomited within an hour (Figure 2). Children were followed up 25–27 days after treatment.

Our inclusion criteria for the baseline analysis was that the children were present for at least 1 day before treatment and so provided a minimum of 2 Kato-Katz thick smears for diagnosis. This gave a sample size of 749 patients. Our inclusion criteria for the efficacy analysis were that the child was (1) present for at least 1 day before and 1 day after treatment; (2) positive for *S. mansoni* at baseline, and (3) successfully treated with praziquantel and albendazole without vomiting. This gave a sample size of 414 patients (Figure 2).

### Sample Egg Reduction Rate

We calculated the sample ERR using

(1) $$\text{Sample ERR}=1-\frac{\text{mean egg count after treatment}}{\text{mean egg count before treatment}},$$

where the mean is calculated at the level of the school using all egg counts, rather than individual-level averages. We used a nonparametric percentile block bootstrap method to calculate confidence intervals (CIs) associated with the sample ERR. Block bootstrap methods [20] account for correlation among observations (egg counts) from the same individual by randomly sampling (with replacement) blocks of data; here, all of an individual's egg counts before and after treatment. We also applied this method to calculate CIs associated with mean egg counts before and after treatment. Details are given in the Supplementary Methods.

### Covariates

The schools were selected to give a variety of different MDA histories: 1 round for Kocoge; an estimated 5 rounds for Bukoba and Bukogabo; and 8 rounds for Musubi and 9 rounds for Bugoto and Bwondha, which we classify as low, medium, and high previous exposure to MDA, respectively (Table 1). While school-level MDA records were not available for Bukoba and Bukagabo, the number of MDA rounds was estimated based on information from Mayuge district officials and district-level MDA coverage from the Ugandan Ministry of Health. The sex and weight of each child were recorded at enrollment. Egg counts for STHs (*Ascaris lumbricoides*, *Trichuris trichiura*, and hookworm species) were also collected. Weight was categorized as low, <22 kg; medium, 22–26 kg, and high, >26 kg. Age and height were recorded, but dropped as covariates due to their correlation with weight. Coinfection with STHs was defined as a binary variable.

### Statistical Analysis

We developed and fitted a statistical model to the *S. mansoni* egg count data in a Bayesian framework using an approach outlined elsewhere [19, 21] and implemented in R [22] with the package MCMCglmm [23]. Full mathematical details are given in Supplementary Methods. Here we give a brief description of the features relevant to the estimation of patient ERRs. The key distributional assumption of the model is that egg counts measured from a patient at a specific time (before or after treatment) are Poisson distributed, with an observation-level random effect permitting extra-Poisson variation or overdispersion [19, 22, 23].

The key systematic component of the model is a log-linear regression structure that describes the change in egg counts after treatment, *x* = 1, compared to before treatment, *x* = 0, in a multiplicative fashion such that the accompanying treatment effect coefficient *β* quantifies the relative change and the ERR is given by 1 – exp(*βx*). Covariates enter the model as fixed effects interacting with *x*, allowing ERRs to vary among patients of different sex, weight category, and MDA exposure. Random-effects coefficients, specific to each patient and school and also interacting with *x*, permit ERRs to vary among patients and among schools sharing the same (fixed effect) covariate values. The correlation (*ρ*) between random effects (at individual-patient or school level) quantifies the association between egg counts before treatment and the treatment effect. A positive correlation at the individual level corresponds to individuals with higher-than-average egg counts at baseline tending to have lower ERRs and vice versa for a negative correlation.

### Ethical Considerations

Approvals were granted by the Uganda National Council of Science and Technology (MoU sections 1.4, 1.5, 1.6) and the Imperial College Research Ethics Committee (EC NO: 03.36. R&D No: 03/SB/033E). Verbal assent was given by every child before inclusion into this study and at school committee meetings comprising of parents, teachers, and community leaders before the onset of the study. Written consent for the children to participate in the study was attained from each head teacher. Participation was voluntary and children could withdraw or be withdrawn from the study at any time. All enrolled children were treated with 40 mg/kg praziquantel and 400 mg albendazole after examination at baseline, and any children who remained positive at follow-up were re-treated.

## RESULTS

### Summary Epidemiological Statistics

Baseline epidemiological data for each of the 6 schools are shown in Table 1. The analysis was performed on 749 children who were present for at least 1 day of sampling before treatment. The prevalence of *S. mansoni* at baseline in the schools surveyed ranged from 44.92% in Bukoba to 94.49% in Musubi. The arithmetic mean eggs per gram (EPG) was calculated by averaging over egg counts from schools and multiplying by a factor of 24, rather than from the fitted model, to be comparable with previous studies in Uganda [24]. The intensity of infection varied 7-fold among schools, from 92 EPG (95% CI, 67–128 EPG) in Bukoba to 742 EPG (95% CI, 518–973 EPG) in Bwondha.

Of the 414 children included in the praziquantel efficacy analysis (Figure 2), 209 (50.48%) were female, and the age range was 6–12 years (median, of 8.5 years). There were no significant differences in the fixed-effect characteristics of included and excluded children (see Supplementary Materials). A total of 4102 egg counts (approximately 10 per child) were collected from Kato-Katz smears: 2090 before treatment and 2012 after treatment. Coinfections of *S. mansoni* and STHs were present in 141 patients (34.06%), of which the majority were hookworm (139 [33.57%]).

### Variation in Praziquantel Efficacy Among Children

The estimated posterior distributions of the model coefficients associated with praziquantel efficacy are summarized in Table 2 as means and 95% Bayesian credible intervals (BCIs). We also present the (Bayesian) *P* values [25] associated with each posterior distribution. Negative coefficients (or their exponent <1) associated with covariate–treatment interactions indicate a lower egg count after treatment and a higher ERR and vice versa for positive coefficients (or their exponent >1). Because the model is specified at the level of the individual patient, coefficients are interpreted as reflecting the fixed covariate effect on a typical child (ie, when the random-effects adjustments are equal to 0) [21].

**Table 2.** Estimated Coefficient Posterior Distributions of the Generalized Linear Mixed Model Fitted to *Schistosoma mansoni* Egg Counts Collected From 414 Children From 6 Primary Schools in Eastern Uganda Before and After Treatment With Praziquantel

Covariate	Category	Coefficient Posterior Mean (95% BCI)	Exponent Coefficient Posterior Mean (95% BCI)	Bayesian *P* Value
Intercept	NA	2.32 (1.36 to 3.25)	10.79 (2.17–21.19)	.01
Treatment	NA	−5.65 (−6.81 to −4.55)	3.91 × 10^−3^ (5.82 × 10^−4^ to 8.86 × 10^−3^)	<.01
MDA exposure	Low (1 round)	−1.33 (−3.20 to 0.48)	0.328 (3.36 × 10^−9^ to 1.03)	.10
	Medium (5 rounds)	−1.42 (−2.94 to 0.25)	0.293 (9.34 × 10^−13^ to 0.793)	.06
Weight	Low (<22 kg)	1.21 (0.40–2.03)	3.57 (1.12–6.72)	<.01
	Medium (22–26 kg)	0.83 (0.01–1.65)	2.45 (0.845–4.74)	.05
Sex	Male	0.13 (−0.48 to 0.78)	1.19 (5.32–1.98)	.67
Coinfection with STH	Positive	0.27 (−0.40 to 0.99)	1.37 (0.569–2.43)	.44

The posteriors are summarized by their mean and 95% BCI. The coefficient estimates all interact with treatment and thus correspond to the effect of the covariate on the egg reduction rate (ERR). Negative coefficients (or their exponent <1) associated with covariate–treatment interactions indicate a lower egg count after treatment and a higher ERR and vice versa for positive coefficients (or their exponent >1) (see “Methods” section).

Abbreviations: BCI, Bayesian credible interval; MDA, mass drug administration; NA, not applicable; STH, soil-transmitted helminth.

The negative coefficients associated with egg counts measured after treatment from different low (*β* = −1.32, *P* = .10) and medium (*β* = −1.42, *P* = .060) MDA exposure categories indicate that a typical child within a school that has received fewer previous MDA rounds has a higher ERR than a typical child in a school that has received more previous rounds of MDA, although these effects are not statistically significant. The positive coefficients associated with medium (*β* = .83, *P* = .0024) and low (*β* = 1.21, *P* = .048) weight categories indicate that a typical child in these weight categories has a lower ERR than a typical child in the high weight category. The coefficients of other covariates fitted as interaction terms in the model (sex and coinfection with STH) are not statistically significantly different from zero. The estimated negative correlation between children's egg counts before treatment and the treatment effect (*ρ* = −0.17; see Supplementary Methods) indicates that children with higher egg counts at baseline tend to have *higher* ERRs, albeit the effect is of only marginal statistical significance (95% BCI, −0.32 to −0.019).

**Figure 3.**
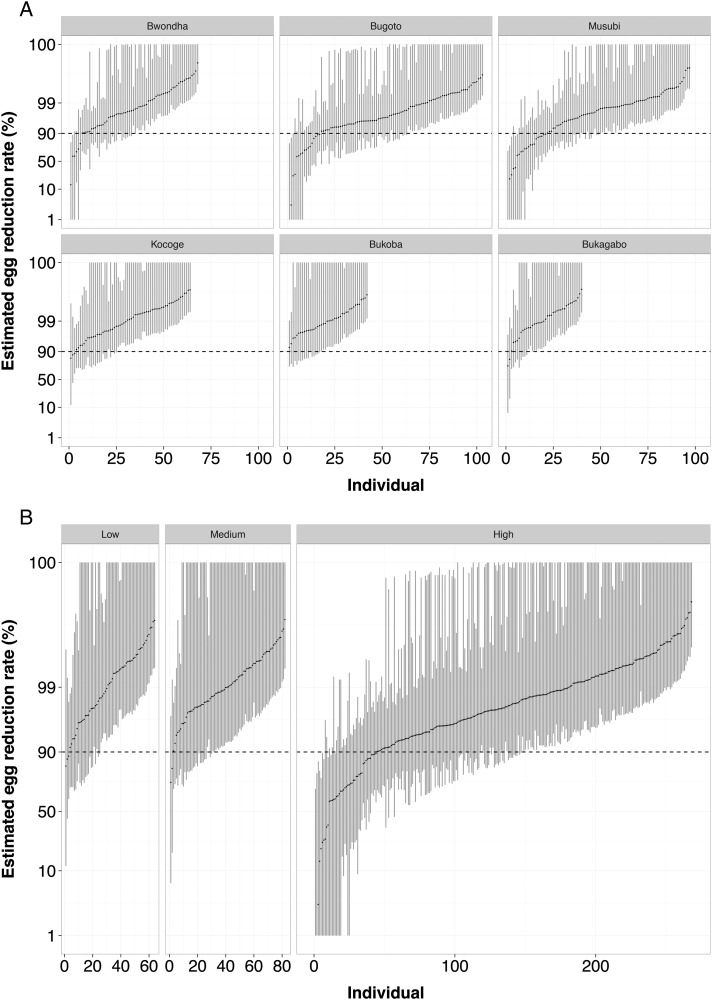
Estimated egg reduction rates of *Schistosoma mansoni* from 414 children in eastern Uganda treated with praziquantel. Individual estimates are stratified by school (*A*) and by mass drug administration (MDA) exposure category (*B*). Black points indicate the mean of the posterior distribution, and the gray bars indicate 95% Bayesian credible intervals (BCIs). The hashed lines correspond to the nominal reference threshold of 90% suggested by the World Health Organization. Note that the BCIs have been truncated at 1% and 99%. Category of MDA reflects the previous number of rounds of treatment with praziquantel the schools have received (high is 8 or 9; medium is 5; low is 1).

Individual children's ERRs are variable within each school and MDA exposure category; however, in the high MDA exposure category, 16.42% (95% BCI, 13.42%–19.78%) of the posterior mean individual ERRs are below the 90% reference efficacy [18] compared with 4.55% (95% BCI, 1.22%–9.76%) and 5.11% (95% BCI, 0.00%–10.94%) in the medium and low categories, respectively (Figure 3). The BCIs quantify the considerable uncertainty in the individual mean ERR estimates and reflect both the variation among egg counts measured from the same child and the number of counts measured per child, which ranged from 4 to 12. The estimated ERRs and associated 95% BCIs for each child, stratified by school and MDA exposure category, are shown in Figure 3.

### Variation in the Efficacy of Praziquantel Among Schools

We marginalized (averaged) over the posterior distributions of the fixed- and random-effects coefficients to yield average ERRs by schools and MDA exposure categories. Average ERRs are generally high and ≥90%; however, we find that schools within the high MDA exposure category show a significantly lower ERR (91.49% [95% BCI, 88.23%–93.64%]) compared with both the medium (98.04% [95% BCI, 96.13%–99.08%]) and low (97.81% [95% BCI, 95.5%–98.96%]) categories, having adjusted for other covariate fixed effects (Table 3). For comparison with the model-derived estimates and for comparability with previous studies, we also calculated sample estimated ERRs. The sample estimated ERRs follow the same pattern as the model-derived ERR estimates, with more intensively treated schools showing lower ERRs, and fall within the corresponding 95% BCI (Table 3). Resampling from the dataset using fewer egg count readings suggests that 3 days of duplicate Kato-Katz readings before and after treatment were necessary to detect the differences in anthelmintic efficacy we observe between MDA exposure categories (see Supplementary Materials).

**Table 3.** Estimated Average Egg Reduction Rates of *Schistosoma mansoni* in 414 Children From 6 Primary Schools in Eastern Uganda Following Treatment With Praziquantel

MDA Exposure Category	No.	Cleared, No. (%)	ERR, %		School	No.	Cleared, No. (%)	ERR, %	
			Sample (95% CI)	Model (95% BCI)				Sample (95% CI)	Model (95% BCI)
High (8–9 rounds)	268	151 (56.34)	92.10 (87.42–95.71)	91.49 (88.23–93.64)	Bwondha	68	40 (58.82)	94.27 (88.13–98.26)	94.19 (90.65–96.34)
					Bugoto	103	62 (60.19)	89.63 (77.65–96.65)	91.20 (85.40–94.50)
					Musubi	97	49 (50.51)	91.98 (82.35–97.69)	89.90 (83.53–93.49)
Medium (5 rounds)^a^	82	68 (82.93)	98.71 (97.50–99.53)	98.04 (96.13–99.08)	Bukoba	42	37 (88.10)	98.98 (97.55–99.85)	98.50 (96.26–99.52)
					Bukagabo	40	31 (77.50)	98.58 (96.50–99.64)	97.56 (94.59–99.01)
Low (1 round)	64	45 (70.31)	98.87 (98.07–99.60)	97.81 (95.51–98.96)	Kocoge	64	45 (70.31)	98.87 (98.07–99.60)	97.81 (95.51–98.96)

Sample estimates are calculated using equation 1 in “Methods” section, sample egg reduction rates, with 95% confidence intervals (CIs) calculated using a block bootstrap approach to account for correlation among egg counts repeatedly measured from the same child. Model estimates and 95% BCIs correspond to the posterior distribution of the average ERR by mass drug administration (MDA) exposure category and by school calculated by marginalizing (averaging) over the appropriate fixed- and random-effects coefficients of the fitted generalized linear mixed model.

Abbreviations: BCI, Bayesian credible interval; ERR, egg reduction rate.

^a^ Estimated from district-level MDA coverage from the Ministry of Health.

**Figure 4.**
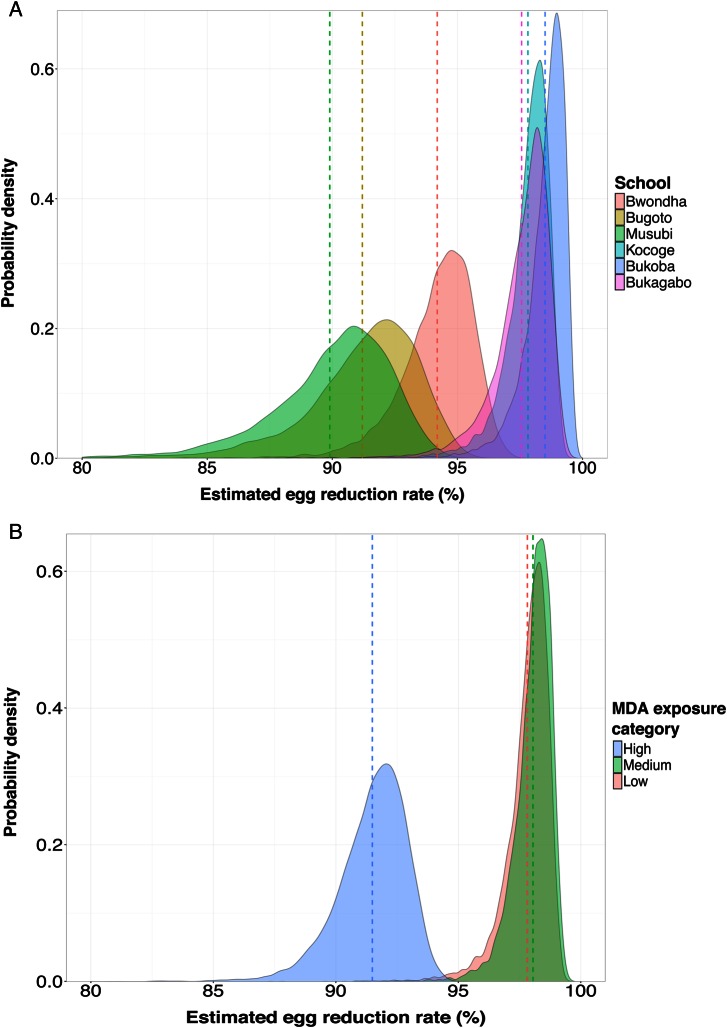
Posterior distribution of average egg reduction rates (ERRs) of *Schistosoma mansoni* in 414 children from 6 primary schools in eastern Uganda treated with praziquantel. The average ERRs are stratified by school (*A*) and by mass drug administration (MDA) exposure category (*B*). The posteriors were constructed by marginalizing over the fixed- and random-effects coefficients estimated from the generalized linear mixed model. The colored hashed lines indicate the mean of the posterior. Category of MDA reflects the previous number of rounds with praziquantel the schools have received (high is 8 or 9; medium is 5; low is 1).

The posterior distributions of average ERRs by school and MDA exposure category are shown in Figure 4. The distribution associated with the high MDA exposure category is separated from the distribution of the medium and low categories, in accordance with a lower average efficacy of praziquantel for children in schools with a higher past exposure to MDA.

## DISCUSSION

We have tested the impact of a school's past exposure to MDA with praziquantel on present treatment outcomes for *S. mansoni*. We have demonstrated through the use of an appropriate statistical modeling technique that treatment efficacy, as quantified by the ERR, is lower in schools that have been treated in 8 or 9 previous MDA rounds compared with other schools from the same or nearby districts that have undergone ≤5 rounds of MDA with praziquantel.

From the baseline data we collected from the schools (Table 1), we found a high prevalence (>84%) and infection intensity (>400 EPG) in Bugoto, Bwondha, and Musubi, the schools that have undergone the highest number of previous MDA rounds. It is surprising that *S. mansoni* infection remains entrenched within these foci despite up to 9 rounds of MDA. We concur that persistence of the parasite population is driven by continued reinfection from transmission hotspots in Lake Victoria [26]. It is concerning for efforts to halt transmission of *S. mansoni* through MDA alone that areas with high infection intensity are so resilient to repeated treatments.

We found that the weight of children was associated with estimated ERRs. The dose of praziquantel is weight-standardized at 40 mg/kg and hence one would not expect differences in drug efficacy among children of different weight. The lower ERR in lighter children suggests that they are not receiving a sufficient dose, perhaps indicating that there is a minimum therapeutic dose that lighter children are failing to receive. There could also be an age-dependent effect (as weight and age are closely correlated) reflecting synergism between the development of protective immune responses and the antischistosomal activity of praziquantel [27].

A recent meta-analysis of praziquantel efficacy reported an average ERR of 89.1% (95% CI, 83.3%–94.2%) among treated *S. mansoni* infections in schoolchildren, with a median follow-up time of 5 weeks [28]. The comparable estimates of the ERR in this study are the average school-level estimates, including those for different MDA exposure categories. None of our estimates extend below the lower CI of the meta-review estimates, suggesting that praziquantel remains broadly efficacious in all our study sites (although heterogeneity in study design will naturally widen CIs estimates from a meta-analysis compared to estimates obtained from a single study). However, of concern is the significant difference in estimated ERRs we observe between schools that have received more previous rounds of MDA compared with those that have received fewer rounds (Table 3 and Figure 4).

Variation in anthelmintic efficacy is driven by parasite- and host-specific factors. Without long-term longitudinal data (ie, on the scale of years, over multiple rounds of MDA), or baseline data from before MDA commenced, we cannot determine whether heterogeneities among ERRs, at either the individual or school level, are static or changing temporally. A systematic decline in ERRs could be indicative of increasing parasite tolerance or emerging resistance to praziquantel [29, 30]. Intervention programs using MDA should incorporate regular assessment of drug efficacy into their monitoring and evaluation protocols.

A recent analysis of praziquantel efficacy against *S. mansoni* found variation among ERRs from individual patients to be greater in Uganda than in other countries [21]. *Schistosoma mansoni* parasites in Uganda have been observed to be more genetically diverse than elsewhere [31], which could be driving more variable treatment outcomes. Host factors such as genetic variation or differences in nutritional status are unlikely to be sufficient to explain the variation in ERRs because of the spatial proximity and similar patterns of subsistence in the schools surveyed (Figure 1). It is also unlikely that different rates of reinfection are driving substantive differences among patient ERRs. This is because the time to follow-up (25–27 days) is short and the statistical model is designed to control for different rates of reinfection by modeling the intensity of infection after treatment relative to the intensity before treatment such that each individual acts as its own control.

We are left with the possible explanation that reduced praziquantel efficacy is caused by drug-tolerant genetic variants within the *S. mansoni* population in highly treated areas. Genetic changes associated with drug resistance have been associated with drug-induced selection pressures in human and veterinary helminthiases [32–35]. Ultimately, drug-induced resistance can only be confirmed by linking changes in resistance allele frequencies to phenotypic indicators of drug tolerance or reduced susceptibility. As neither the precise mode of action of praziquantel nor the genetic basis of resistance in schistosomes is completely understood [36], the powerful approaches offered by next-generation sequencing will be essential to scan for selection across whole genomes [37, 38]. Such approaches would help to establish if parasite genetic variation could be driving the degree of variation in praziquantel efficacy that we have observed in this study.

## Supplementary Material

Supplementary Data

Supplementary Data
