# Supplementary material for: Reduced Efficacy of Praziquantel Against Schistosoma mansoni Is Associated With Multiple Rounds of Mass Drug Administration
Source: Clin Infect Dis. 2016 Jul 28;63(9):1151–9. doi: 10.1093/cid/ciw506 (PMC5064161; doi:10.1093/cid/ciw506)
Supplement: Supplementary Data [file ciw506_Supplementary_Data.zip › ciw506supp.docx]

**Title**

Reduced efficacy of praziquantel against *Schistosoma mansoni* associated with multiple-rounds of mass drug administration

**Authors**

Thomas Crellen, Martin Walker, Poppy H.L. Lamberton, Narcis B. Kabatereine, Edridah M. Tukahebwa, James A. Cotton, Joanne P. Webster

**Supplementary Material**

**Supplementary Methods**

**Percentile Block Bootstrap**

The percentile block bootstrap approach [1] to calculate 95% confidence intervals (CIs) associated with sample egg reduction rates (ERRs) was implemented as follows:

1. Sample with replacement *n* vectors (collections) of egg counts measured before *and* after treatment from individual children.
2. Calculate the sample ERR from the new sample of egg counts using Equation (1) in the main text.
3. Repeat steps (i) and (i) 10,000 times to yield an empirical sampling distribution of the (sample) ERR.
4. Calculate CIs from the 2.5% and 97.5% percentiles of the (sample) ERR sampling distribution.

**Generalized Linear Mixed Model**

We defined a generalized linear mixed model of the form

*y_ijk_*~ Poisson(*μ_ijk_*),

**μ***_jk_* = exp(**βX**^T^*_j_* + **b***_j_***Z**^T^*_j_* + **b***_k_***Z**^T^*_k_* + **e***_jk_*),

**b***_j_* ~ MVN(**0**, **Σ**_ID_),

**b***_k_* ~ MVN(**0**, **Σ**_SC_),

*e_ijk_* ~ N(0,*σ*^2^). (1)

Here, *y_ijk_* denotes an *Schistosoma mansoni* egg count *i* measured from individual *j* from school *k* that is assumed to be a realization from a Poisson a distribution with mean *μ_ijk_*. Each *μ_ijk_* corresponds to an element within the vector **μ***_jk_* = {*μ*_1_*_jk_*, *μ*_2_*_jk_*,…, *μ_njk_*} that is *conditional on* the fixed effect covariates within matrix **X***_j_* and accompanying coefficients within vector **β**= {*β* **_0_**,*β***_1_**,…, *β_m_*}, and on the individual- and school-level random effects within **Z***_j_* and **Z***_k_* and the accompanying coefficients **b***_j_* = {*b­*_0_*_j_*, *b*_1_*_j_*} and **b***_k_* = {*b­*_0_*_k_*, *b*_1_*_k_*}. The fixed effects within **X***_j_* comprise indicator variables for mass drug administration category, weight category, sex and presence of soil-transmitted helminth co-infection interacting with a binary indicator of whether an egg count was measured before or after treatment. The random effects covariates within **Z***_i_* and **Z***_k_* include intercept terms specific to individual *j* and and school *k* in addition to school- and individual-specific ‘gradient’ terms for the effect of treatment. The random effects coefficients **b***_j_* and **b***_k_* follow a multivariate normal distribution (MVN) with means equal to zero and a variance-covariance matrix **Σ**_ID_ and **Σ**_SC_ respectively. The model also includes an additional vector of random effects, **e***_ik_*, which are specific to each observation and permit extra-Poisson variation among egg counts measured from the same individual at the same time point [2-4]. Each *e_ijk_* is assumed normally distributed (on the logarithmic scale) with mean 0 and variance *σ*^2^, which quantifies the degree of overdispersion.

The logarithmic relationship between *μ_ijk_*  and the fixed and random effects in (1) ensures that coefficients associated with egg counts measured after treatment, so-called treatment effects, quantify multiplicative relative changes compared to before treatment. The covariance terms within **Σ**_ID_ and **Σ**_SC_ defined in model (1) quantify the association between egg counts before treatment and the treatment effect. The first diagonal element of these matrices, *σ*_0_^2^, quantifies variation in egg counts among individuals or schools before and after treatment, capturing both unit level clustering and longitudinal correlation. The second diagonal element, *σ*_1_^2^, quantifies variation in the treatment effect (i.e. variation among ERRs). Hence, the off-diagonal covariance terms—which we express in scaled form as a correlation coefficient, *ρ*—quantify how egg counts before treatment and the treatment effect vary together. For example, a positive correlation indicates that higher than average egg counts (before *and* after treatment) are associated with less negative values of the treatment effect coefficient, corresponding to smaller relative changes (after treatment compared to before treatment) and hence lower ERRs. For example, a positive correlation at the patient level, corresponds to individuals with high egg counts (heavy infections) tending to have *lower* ERRs following treatment. A negative correlation indicates the opposite, of individuals with high egg counts tending to have *higher* ERRs following treatment.

We fitted the model (1) to the data on egg counts in a Bayesian framework using Markov chain Monte Carlo techniques [2,5]. We defined uninformative / vague parameter prior distributions and ran the Markov chains for 100,000 iterations with a 2,500 iteration burn-in, yielding an effective sample size of >1000 for all parameters and hyperparameters. We visually checked that the chains had converged to a stable posterior distribution. We constructed posterior distributions for ERRs associated with schools and MDA exposure categories by marginalizing over the appropriate subset of the coefficients. The analysis was performed using R version 3.0 [6] and the package MCMCglmm version 2.21[4]. We provide the R code in the Supplementary Information, including the custom function for estimating the marginal posteriors of the ERRs by school and treatment category.

**Sensitivity of ERR estimates to number of readings taken**

We assessed the sensitivity of our results to the number of readings per individual by sub-sampling from the original dataset between 1-6 readings before and 1-6 readings after treatment and using the same mixed effects model to calculate the estimated ERR for the “MDA exposure group” for each school. Note that the readings refer to sequential time points (rather than random resampling) so that, for instance, a child may be absent for readings 1 and 2 after treatment but present for readings 3-6. This gives an indication of what the results would show had we taken fewer egg counts and/or sampled on fewer days. The results, as included in the revised Supplementary Information, are shown below where n indicates the number of readings (before and after treatment). The estimated ERR calculated using all readings (up to 6 before and after) as reported in the main text is also included for comparison.

In all instances the ERR observed in the “High” MDA exposure schools remains the lowest. However, a significant difference (i.e. non-overlapping 95% credible intervals) between MDA exposure groups is only observed with 5 or 6 readings. This result indicates that 3 days of Kato-Katz readings (2 slides made per day) was necessary to detect the difference in ERR between schools.

Reading 1 before and after (*n*= 678)

| Treatment intensity group | Mean ERR (%) | 95% BCI |
| --- | --- | --- |
| High | 95.97 | 87.31, 98.87 |
| Medium | 98.28 | 92.61, 99.76 |
| Low | 99.13 | 95.74, 99.95 |

Readings 1 and 2 before and after (*n* = 1356)

| Treatment intensity group | Mean ERR (%) | 95% BCI |
| --- | --- | --- |
| High | 89.75 | 80.43, 94.16 |
| Medium | 94.77 | 81.67, 98.60 |
| Low | 96.36 | 84.43, 99.54 |

Readings 1, 2 and 3 before and after (*n* = 2130)

| Treatment intensity group | Mean ERR (%) | 95% BCI |
| --- | --- | --- |
| High | 86.31 | 72.44, 92.39 |
| Medium | 97.17 | 93.70, 98.81 |
| Low | 96.48 | 88.37, 99.07 |

Readings 1, 2, 3 and 4 before and after (*n* = 2904)

| Treatment intensity group | Mean ERR (%) | 95% BCI |
| --- | --- | --- |
| High | 86.89 | 77.20, 91.78 |
| Medium | 97.76 | 95.54, 98.94 |
| Low | 94.34 | 81.91, 98.41 |

Readings 1, 2, 3, 4 and 5 before and after (*n* = 3503)

| Treatment intensity group | Mean ERR (%) | 95% BCI |
| --- | --- | --- |
| High | 89.64 | 83.80, 92.92 |
| Medium | 97.95 | 95.84, 99.04 |
| Low | 97.47 | 94.55, 98.86 |

Readings 1, 2, 3, 4, 5 and 6 before and after (*n*= 4102)

| Treatment intensity group | Mean ERR (%) | 95% BCI |
| --- | --- | --- |
| High | 91.49 | 88.23, 93.64 |
| Medium | 98.04 | 96.13, 99.08 |
| Low | 97.81 | 95.51, 98.96 |

**Representativeness of the included sample for ERR analysis**

Of the children who were registered and positive for *S. mansoni* (n = 542), 414 were included in the efficacy analysis. We have compared the fixed effect characteristics (sex, age, weight category, treatment intensity category) of the 414 children included in the efficacy analysis with the 128 excluded children, finding no statistically significant differences. We include these comparisons in the revised Supplementary Information and below.

**Sex**

|  | Included | Excluded |
| --- | --- | --- |
| Male | 205 | 74 |
| Female | 209 | 54 |

Chi-Square test of association

X-squared = 2.3718, df = 1, p-value = 0.1235

**Age**

|  | Included | Excluded |
| --- | --- | --- |
| 6 | 57 | 19 |
| 7 | 68 | 23 |
| 8 | 82 | 22 |
| 9 | 72 | 12 |
| 10 | 54 | 24 |
| 11 | 66 | 17 |
| 12 | 15 | 11 |

Chi-Square test of association

X-squared = 12.323, df = 6, p-value = 0.05514

**Weight Category**

|  | Included | Excluded |
| --- | --- | --- |
| High | 109 | 29 |
| Medium | 141 | 34 |
| Low | 164 | 46 |

Chi-Square test of association

X-squared = 0.35817, df = 2, p-value = 0.836

**MDA Exposure Category**

|  | Included | Excluded |
| --- | --- | --- |
| High | 268 | 83 |
| Medium | 82 | 28 |
| Low | 64 | 17 |

Chi-Square test of association

X-squared = 0.51651, df = 2, p-value = 0.7724

**Supplementary Tables and Figures**

**Supplementary Table 1.** **Sample estimates of the prevalence and intensity of *Schistosoma mansoni* infection in children from six primary schools in eastern Uganda before treatment with praziquantel.** The number of previous rounds of Mass Drug Administration (MDA) the school has been exposed and corresponding MDA Exposure Categories are shown. Infection intensity is quantified as the arithmetic mean number of egg per gram of faeces (EPG). Confidence intervals (CIs) are calculated using a block bootstrap approach to account for correlation among egg counts repeatedly measured from the same child. This table includes GPS coordinates and elevation of the schools included in the study.

**Supplementary Table 2. Estimated coefficient posterior distributions of the generalized linear mixed model fitted to *Schistosoma mansoni* egg counts collected from 414 children from six primary schools in eastern Uganda before and after treatment with praziquantel.** The posteriors are summarised by their mean and 95% Bayesian credible interval (BCI). The coefficients estimates with ‘before treatment’ written in parenthesis correspond to the effect of the covariate on baseline egg counts. Coefficient estimates with ‘after treatment’ written in parentheses corresponds to the effect of the covariate on the egg reduction rate (ERR); a positive value indicates a *lower* ERR (compared to the reference category) and a negative value indicates a *higher* ERR (see Methods).

**Supplementary Figure 1. Egg counts before treatment as estimated by the generalized linear mixed model for each individual (*n*= 414).** Black points indicate the mean of the posterior distribution and the grey bars indicate 95% Bayesian credible intervals (BCIs).

**Supplementary Figure 2. Egg counts after treatment as estimated by the generalized linear mixed model for each individual (*n*= 414).** Black points indicate the mean of the posterior distribution and the grey bars indicate 95% Bayesian credible intervals (BCIs).

**Supplementary Table 1.**

| **School** | **District** | **MDA**  **Exposure**  **Category** | **GPS North** | **GPS East** | **Elevation (m)** | **Recruited** | **Tested at Baseline** | **Positive for**  ***S. mansoni***  **(%)** | **Mean EPG**  **(95 % CI)** |
| --- | --- | --- | --- | --- | --- | --- | --- | --- | --- |
| Bwondha | Mayuge | High  (9 rounds) | 0.17775 | 33.56138 | 1150 | 98 | 96 | 87  (90.63) | 742  (518-973) |
| Bugoto | Mayuge | High  (9 rounds) | 0.32369 | 33.62837 | 1140 | 183 | 170 | 144  (84.71) | 436  (339-534) |
| Musubi | Mayuge | High  (8 rounds) | 0.31105 | 33.6652 | 1155 | 128 | 127 | 120  (94.49) | 465  (362-600) |
| Bukoba | Mayuge | Medium  (5 rounds)^a^ | 0.32079 | 33.50797 | 1140 | 120 | 118 | 53  (44.92) | 92  (67-128) |
| Bukagabo | Mayuge | Medium  (5 rounds)^a^ | 0.23397 | 33.4646 | 1165 | 120 | 118 | 57  (48.31) | 306  (197-442) |
| Kocoge | Tororo | Low  (1 round) | 0.77552 | 34.23175 | 1269 | 120 | 120 | 81  (67.50) | 347  (245-456) |
| **Total** |  |  |  |  |  | 769 | 749 | 542  (72.36) | 382  (338-446) |

^a^ Estimated from district-level MDA coverage from Ministry of Health

| **Covariate** | **Category** | **Coefficient posterior mean (95% BCI)** | **Exponent coefficient posterior mean (95% BCI)** | **Bayesian *p* value** |
| --- | --- | --- | --- | --- |
| Intercept | NA^a^ | 2.32 (1.36, 3.25) | 10.79 (2.17, 21.19) | 0.01 |
| Treatment | After treatment | -5.65 (-6.81, -4.55) | 3.91x10^-3^ (5.82x10^-4^, 8.86x10^-3^) | <0.01 |
| MDA^b^ exposure (before treatment) | Low (1 round) | -0.06 (-1.98, 1.65) | 1.09 (2.10x10^-15^, 3.15) | 0.87 |
|  | Medium (5 rounds) | -0.73 (-2.13, 0.68) | 0.531 (2.81x10^-7^, 1.29) | 0.16 |
| Weight (before treatment) | Low (<22kg) | -0.74 (-1.13, -0.31) | 0.487 (0.307, 0.708) | <0.001 |
|  | Medium (22-26kg) | -0.23 (-0.64, 0.19) | 0.808 (0.500, 1.62) | 0.29 |
| Sex (before treatment) | Male | -0.12 (-0.43, 0.22) | 0.897 (0.629, 1.21) | 0.46 |
| Co-infection with STH^c^ (before treatment) | Positive | -0.24 (-0.57, 0.10) | 0.796 (0.543, 1.08) | 0.17 |
| MDA exposure (after treatment) | Low (1 round) | -1.33 (-3.20, 0.48) | 0.328 (3.36x10^-9^, 1.03) | 0.10 |
|  | Medium (5 rounds) | -1.42 (-2.94, 0.25) | 0.293 (9.34x10^-13^, 0.793) | 0.06 |
| Weight (after treatment) | Low (<22kg) | 1.21 (0.40, 2.03) | 3.57 (1.12, 6.72) | <0.01 |
|  | Medium (22-26kg) | 0.83 (0.01, 1.65) | 2.45 (0.845, 4.74) | 0.05 |
| Sex (after treatment) | Male | 0.13 (-0.48, 0.78) | 1.19 (5.32, 1.98) | 0.67 |
| Co-infection with STH (after treatment) | Positive | 0.27 (-0.40, 0.99) | 1.37 (0.569, 2.43) | 0.44 |

**Supplementary Table 2.**

**Supplementary Figure 1.**

**Supplementary Figure 2.**

**Supplementary References**

1. Lahiri SN. Resampling Methods for Dependent Data. New York: Springer, **2003.**
2. Walker M, Churcher TS, Basáñez M-G. Models for measuring anthelmintic drug efficacy for parasitologists. Trends Parasitol **2014**; 30(11): 528-37.
3. Elston D, Moss R, Boulinier T, Arrowsmith C, Lambin X. Analysis of aggregation, a worked example: numbers of ticks on red grouse chicks. Parasitology **2001**; 122(05): 563-9.
4. Hadfield JD. MCMC methods for multi-response generalized linear mixed models: the MCMCglmm R package. Journal of Statistical Software **2010**; 33(2): 1-22
5. Walker M, Mabud TS, Olliaro PL, et al. New approaches to measuring anthelminthic drug efficacy: parasitological responses of childhood schistosome infections to treatment with praziquantel. Parasit Vectors **2016**; 9(1): 1.
6. R Core Team. R: A Language and Environment for Statistical Computing.  Vienna: R Foundation for Statistical Computing, **2015**.
