# Supplementary material for: Reduced Efficacy of Praziquantel Against Schistosoma mansoni Is Associated With Multiple Rounds of Mass Drug Administration
Source: Clin Infect Dis. 2016 Jul 28;63(9):1151–9. doi: 10.1093/cid/ciw506 (PMC5064161; doi:10.1093/cid/ciw506)
Supplement: Supplementary Data [file supp_63_9_1151__index.html]

Reduced efficacy of praziquantel against Schistosoma mansoni is associated with multiple-rounds of mass drug administration — Reduced Efficacy of Praziquantel Against Schistosoma mansoni Is Associated With Multiple Rounds of Mass Drug Administration — Reduced Efficacy of Praziquantel Against Schistosoma mansoni Is Associated With Multiple Rounds of Mass Drug Administration — Supplementary Data 

# Reduced Efficacy of Praziquantel Against *Schistosoma mansoni* Is Associated With Multiple Rounds of Mass Drug Administration

## Supplementary Data

Supplementary Data

- Supplementary Data - Docx file
